# Correlation between visceral adiposity index and erectile dysfunction in American adult males: a cross-sectional study based on NHANES

**DOI:** 10.3389/fendo.2023.1301284

**Published:** 2023-12-06

**Authors:** Mingming Xu, Hang Zhou, Ruihao Zhang, Yang Pan, Xiaoqiang Liu

**Affiliations:** ^1^ Department of Urology, Tianjin Medical University General Hospital, Tianjin, China; ^2^ Department of Lung Cancer Surgery, Tianjin Medical University General Hospital, Tianjin, China

**Keywords:** erectile dysfunction, visceral adiposity index, NHANES, cross-sectional study, male

## Abstract

**Background:**

The risk of visceral obesity on erectile function has recently attracted much attention. The visceral adiposity index (VAI) is a brief and reliable indicator of visceral obesity measurement. Nevertheless, the association between VAI and erectile dysfunction (ED) is not completely clarified.

**Methods:**

Data from NHANES 2001–2004 were enrolled in this study. Erectile function was assessed by a database-self-administered questionnaire. VAI was calculated with body mass index (BMI), waist circumference (WC), triglyceride (TG), and high-density lipoprotein (HDL) cholesterol. The weighted logistic regression model was performed to evaluate the association between VAI and ED.

**Results:**

Ultimately, 3380 participants were enrolled in the study, including 900 with ED and 2480 without ED. Compared to participants without ED, those with ED generally had higher levels of VAI (1.76 vs. 1.53). The weighted logistic regression analyses demonstrated increased odds of developing ED in participants within the 4th quartile (Q4) of VAI compared to the 1st quartile (Q1) of VAI (OR = 2.023; 95% CI, 1.534–2.669; *P* < 0.001). Similar results were still obtained after adjusting for the relevant covariates (OR = 1.404; 95% CI, 1.008–1.954; *P* = 0.044). In subgroup analyses grouped by smoking status, higher VAI was associated with increased odds of developing ED only in the current smoking group (OR = 1.092; 95% CI, 1.021–1.167; *P* = 0.010).

**Conclusion:**

This study indicated that higher VAI is independently related to ED risk and that early intervention is necessary to reduce the progression of ED with high VAI levels.

## Introduction

Erectile dysfunction (ED) is the inability to attain and sustain an erection that is sufficiently satisfactory (1, 2). It is an extremely prevalent male disease that usually affects males over the age of 40 (1). It is estimated that about 322 million males will suffer from it by 2025 (3). The mechanism of ED is complex, involving the psychological, neurological, hormonal, and vascular systems. Endothelial dysfunction and sex hormone abnormalities are two of the most prominent aspects (4, 5). Old age, inadequate physical activity, abnormal lipid profiles, and obesity are strongly related to ED (6, 7). Some studies have shown that patients with ED normally have a higher waist circumference (WC) (8). ED is not only a sexual dysfunction but also has a potential association with cardiovascular disease. A large amount of evidence supports the view that ED may be an early predictor of cardiovascular disease (9).

The visceral adiposity index (VAI) has gained significant popularity as a precise and widely used index for assessing the distribution and function of visceral fat in contemporary times (10). It stands out as an innovative index, as it combines both physical measurements and metabolic markers. The determination of VAI necessitates the integration of body mass index (BMI), waist circumference (WC), triglyceride (TG), and high-density lipoprotein (HDL) cholesterol by means of a mathematical equation (11). Amato et al. demonstrated that an increase in VAI was tightly tied to cardiometabolism. Furthermore, a large cohort study from Beijing, China, suggested that VAI was an independent risk factor for diabetic nephropathy in the Chinese population (12). In addition, a considerable body of research has shown that higher VAI is positively associated with female sexual dysfunction (13, 14).

Several previous studies have analyzed the association between VAI and ED (15, 16). The findings showed that the mean VAI of the ED group was greater than that of the non-ED group. However, the number of participants in these studies was extremely small. Therefore, this study was further analyzed with data from a large sample of the National Health and Nutrition Examination Survey (NHANES). And hopefully, more reliable evidence between VAI and ED will be available.

## Materials and methods

### Population analyzed in this study

**Figure 1:**
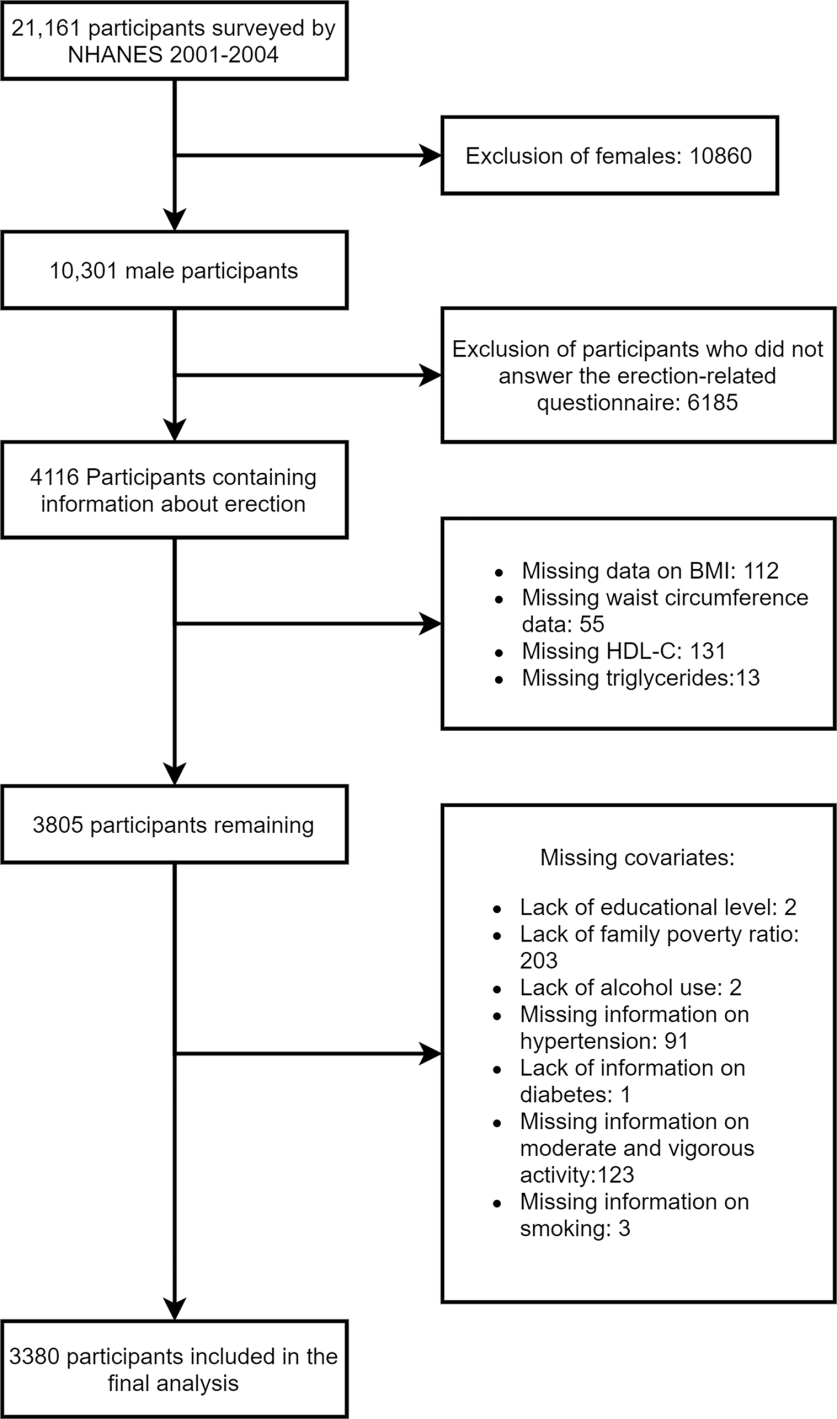
Research flow chart for the present study.

The National Health and Nutrition Examination Survey (NHANES) is a multifaceted study undertaken by the U.S. government to comprehensively assess the health and nutritional status of the population (17). The wealth of data obtained through NHANES serves as a crucial resource for researchers and healthcare professionals, empowering them to make well-informed decisions pertaining to public health and nutrition. Because the NHANES database is publicly accessible, no additional ethics are needed. NHANES data from 2001–2004 were used in this study because only these two survey cycles contained the information from the ED questionnaire. Participants with missing information on ED, VAI, and related covariates were removed from this study, and the detailed process was described in the flowchart (**Figure 1**).

### Measurement of visceral adiposity index

VAI was the main exposure factor in this study. It was calculated by combining waist circumference, BMI, triglycerides, and HDL. For males, the formula for its calculation was as follows: (WC/(39.68+(1.88×BMI))) × (TG/1.03) × (1.31/HDL).

### Definition of erectile dysfunction

In NHANES data, EDs were typically ascertained through a self-report format. Specifically, it was assessed via questions in a questionnaire: How would you describe your ability to get and keep an erection adequate for satisfactory intercourse? Participants who answered “sometimes able” or “never able” were usually considered to be ED.

### Other clinical characteristics

To further assess the association between VAI and ED, this study adjusted for nine relevant covariates: age, diabetes, hypertension, smoking, moderate physical activity, vigorous physical activity, alcohol use, education level, and family poverty ratio. Hypertension was operationally defined as meeting one of the following criteria: systolic blood pressure equal to or exceeding 140 mmHg, diastolic blood pressure equal to or exceeding 90 mmHg, or a self-reported diagnosis of hypertension by medical personnel. Diabetes was assessed based on a questionnaire. In the past 30 days, having done any vigorous activity for at least 10 minutes was defined as vigorous activity. similarly seen in moderate activity. Alcohol use was defined as the consumption of a minimum of 12 alcoholic beverages within a one-year period.

### Statistical analysis

Continuous variables were commonly expressed as the mean (standard deviation, SD) or median (interquartile range, IQR). Categorical variables were then expressed in terms of numbers (percentage). The t-test, Mann-Whitney U test, and chi-square test were performed to compare the analysis between the different groups. In the context of statistical analysis, a p-value that is lower than 0.05 is deemed to possess significant statistical relevance. To further analyze the association between VAI and ED, univariate and multivariate logistic regression models were constructed in this study. Finally, all analyses in this study were based on SPSS 26 and Stata 15 software.

## Results

In the final analysis, a total of 3380 participants were included, with comprehensive details of all individuals provided in **Table 1**. Notably, the group with ED exhibited a significantly higher age compared to the group without ED (68.00 vs. 42.00). Moreover, a relatively elevated proportion of participants within the ED group displayed comorbidities such as hypertension, diabetes, and physical inactivity when compared to their counterparts without ED (**Table 1**). Furthermore, the ED group demonstrated a higher VAI compared to the non-ED group (1.76 vs. 1.53) (**Table 1**).

**Table 1:** Demographic features of the participants in this study.

	ED		*P* value
	Yes	No	
**Participants (n)**	900	2480	
**VAI, median (IQR)**	1.76 (1.93)	1.53 (1.71)	< 0.001
**Age, median (IQR)**	68.00 (18)	42.00 (22)	< 0.001
**Alcohol use, n (%)**			0.023
yes	728 (80.9)	2088 (84.2)	
no	172 (19.1)	392 (15.8)	
**Hypertension, n (%)**			< 0.001
yes	571 (63.4)	792 (31.9)	
no	329 (36.6)	1688 (68.1)	
**Smoking, n (%)**			< 0.001
never	267 (29.7)	1112 (44.8)	
former	446 (49.6)	637 (25.7)	
current	187 (20.8)	731 (29.5)	
**Diabetes, n (%)**			< 0.001
yes	208 (23.1)	126 (5.1)	
no	692 (76.9)	2354 (94.9)	
**Family poverty ratio, n (%)**			< 0.001
<1.3	236 (26.2)	538 (21.7)	
1.3-3.5	391 (43.4)	919 (37.1)	
≥3.5	273 (30.3)	1023 (41.2)	
**Level of education, n (%)**			< 0.001
less than high school	352 (39.1)	551 (22.2)	
high school	190 (21.1)	644 (26.0)	
higher than high school	358 (39.8)	1285 (51.8)	
**Vigorous activity**			< 0.001
yes	169 (18.8)	1031 (41.6)	
no	731 (81.2)	1449 (58.4)	
**Moderate activity**			< 0.001
yes	415 (46.1)	1324 (53.4)	
no	485 (53.9)	1156 (46.6)	

ED, erectile dysfunction; VAI, visceral adiposity index; IQR, interquartile range.

In univariate analysis, the present study observed increased odds of developing ED in participants within the 4th quartile (Q4) of VAI compared to the 1st quartile (Q1) of VAI (OR = 2.023; 95% CI, 1.534–2.669; *P* < 0.001). Even after adjusting for pertinent covariates, the observed findings persisted, and all of them demonstrated statistically significant associations (OR = 1.404; 95% CI, 1.008–1.954; *P* = 0.044) (**Table 2**).

**Table 2:** Association between VAI and ED.

Variable	Univariable	*P* value	Multivariable	*P* value
	OR (95%CI)		OR (95%CI)	
**VAI**				
**Q1 (≤ 0.921)**	Ref		Ref	
**Q2 (0.921-1.592)**	1.598 (1.203-2.122)	0.001	1.197 (0.849-1.689)	0.305
**Q3 (1.592-2.686)**	1.724 (1.302-2.283)	< 0.001	1.147 (0.820-1.606)	0.423
**Q4 (> 2.686)**	2.023 (1.534-2.669)	< 0.001	1.404 (1.008-1.954)	0.044

ED, erectile dysfunction; VAI, visceral adiposity index; OR, odd ratio.

Univariable, not adjusted; Multivariable, adjusted for age, alcohol use, hypertension, smoking, diabetes, family poverty ratio, level of education, vigorous activity, moderate activity.

In subgroup analyses grouped by smoking status, higher VAI was associated with increased odds of developing ED only in the current smoking group (**Table 3**). In addition, grouping by family poverty ratio, we found that VAI was associated with ED and was statistically significant in the group with a family poverty ratio greater than or equal to 3.5 (**Table 3**).

**Table 3:** Subgroup analysis.

	OR	95%CI	*P* value
Family poverty ratio			
<1.3	1.000	0.969-1.032	0.992
1.3-3.5	0.988	0.946-1.032	0.585
≥3.5	1.065	1.006-1.127	0.030
Smoking			
never	1.004	0.931-1.083	0.913
former	0.976	0.936-1.018	0.257
current	1.092	1.021-1.167	0.010

OR, odd ratio.

## Discussion

Visceral obesity represents a form of central obesity that is more hazardous. A large body of evidence suggests that visceral obesity increases the risk of cardiovascular disease to some extent. Some studies have shown that visceral obesity predisposes to venous thrombosis (18). In addition, a study from Turkey showed that visceral obesity is strongly associated with poor semen parameters (19). Visceral fat area (VFA) and VAI are key indicators for assessing visceral obesity (20). However, VFA is more complex and costly to measure, making VAI a better choice. VAI is widely used as a reliable indicator of visceral obesity to explore the relationship between disease risk. However, the association between VAI and ED remains to be investigated.

This study analyzed the relationship between VAI and ED in the US population on the basis of data from NHANES. For the current analysis, we observed that VAI was higher in the ED group than in the non-ED group. The current findings align with the outcomes of prior research investigations (15). In addition, the regression results showed that higher VAI was associated with increased odds of having ED, and similar findings remained after fully adjusting for covariates. These results indicate that VAI is probably an independent risk factor for ED.

However, the underlying mechanisms of VAI and ED remained to be investigated, but several explanations might exist. VAI is a reliable indicator of visceral fat accumulation and functional metabolic dysfunction. Visceral obesity has been shown to lead to endothelial dysfunction. And a randomized controlled trial in the United States demonstrated that increased visceral fat can lead to endothelial dysfunction even in a healthy young population of normal weight (21). Weight loss would improve the situation to a greater extent. In addition, animal experiments have confirmed that bariatric surgery improves endothelial function by attenuating IFNγ-mediated inflammation in visceral fat (22). In parallel to the effects on endothelial function, obesity typically provokes a decrease in testosterone levels, which may be associated with hypothalamic-pituitary-testicular (HPT) axis suppression and insulin resistance (23). Furthermore, Sebo et al. found a significant increase in adiposity in denuded mice, and when supplemented with testosterone, the increase in visceral fat was inhibited and fat distribution was regulated (24). Caretta et al. (25) found a strong negative correlation between VAI and TT levels and that high VAI was strongly associated with hypogonadism in men with type 2 diabetes. At the same time, dyslipidemia may cause damage to peripheral nerves, of which diabetic peripheral neuropathy is a classic example (26). Not only can dyslipidemia lead to nerve damage, but it can also contribute to ED by damaging smooth muscle cells (27).

Although a previous cross-sectional study initially confirmed that high VAI was independently associated with ED, that study had a particularly small sample size and did not adjust for major confounders, such as diabetes or physical activity (16). Hence, a larger sample of data is required for further analysis. And the present study precisely fills in the shortcomings of the previous study.

Specifically, this study has the following strengths. Firstly, our study was based on the data in NHANES, which has the advantage of a large sample size. Moreover, we adjusted for relevant covariates and performed subgroup analysis, which made the results of this study more robust. However, this study still had a few limitations. First, the determination of ED in this study was based on self-report, which was also a limitation of this database. Second, although we adjusted for covariates to the extent possible, there may be factors that were not controlled for.

## Conclusion

In conclusion, this study indicated that higher VAI is independently related to ED risk and that early intervention is necessary to reduce the progression of ED with high VAI levels.

## Data availability statement

The original contributions presented in the study are included in the article/supplementary material. Further inquiries can be directed to the corresponding author.

## Ethics statement

The studies involving humans were approved by NCHS Research Ethics Review Board. The studies were conducted in accordance with the local legislation and institutional requirements. The participants provided their written informed consent to participate in this study.

## Author contributions

MX: Conceptualization, Methodology, Writing – original draft. HZ: Data curation, Methodology, Writing – review & editing. ZR: Methodology, Writing – review & editing. YP: Investigation, Methodology, Writing – review & editing. XL: Supervision, Validation, Writing – review & editing.
